# Theoretical and Experimental Analysis of Thin-Walled Curved Rectangular Box Beam under In-Plane Bending

**DOI:** 10.1155/2021/8867142

**Published:** 2021-04-30

**Authors:** Long Yanze, Zhang Ke, Shi Huaitao, Li Songhua, Zhang Xiaochen

**Affiliations:** School of Mechanical Engineering, Shenyang Jianzhu University, Shenyang 110168, China

## Abstract

Thin-walled curved box beam structures especially rectangular members are widely used in mechanical and architectural structures and other engineering fields because of their high strength-to-weight ratios. In this paper, we present experimental and theoretical analysis methods for the static analysis of thin-walled curved rectangular-box beams under in-plane bending based on 11 feature deformation modes. As to the numerical investigations, we explored the convergence and accuracy analysis by normal finite element analysis, higher-order assumed strain plane element, deep collocation method element, and inverse finite element method, respectively. The out-of-plane and in-plane characteristic deformation vector modes derived by the theoretical formula are superimposed by transforming the axial, tangential, and the normal deformation values into scalar tensile and compression amounts. A one-dimensional deformation experimental test theory is first proposed, formulating the specific contributions of various deformation modes. In this way, the magnitude and trend of the influence of each low-order deformation mode on the distortion and warping in the actual deformation are determined, and the significance of distortion and warping in the actual curved beams subjected to the in-plane loads is verified. This study strengthens the deformation theory of rectangular box-type thin-walled curved beams under in-plane bending, thus providing a reference for analyzing the mechanical properties of curved-beam structures.

## 1. Introduction

Because of their high strength-to-weight ratios, the curved beam members and particularly those with rectangular cross-sections are widely used in mechanical engineering and building structures. This geometry is the first choice for engineering applications when working in the direction of a fixed load. However, studies of the accuracy of the strength and deformation of thin-walled curved beams have mainly considered the distortion and warping of thin-walled curved beam structures and the complexity of other factors, causing difficulties in obtaining the mechanical properties of thin-walled curved beam structures. The earliest research on thin-walled beams [1] began with Vlasov, while Dabrowski expanded into thin-walled curved beam theory [2]. The analysis of the beam by Y.Y. Kim and J.H. Kim (1999,2000) is accurate in that deformation is mainly caused by distortion and warping [3, 4], which increases the accuracy of the analysis of the curved beam. Y. Kim and Y.Y. Kim analyzed the one-dimensional high-order theory of the in-plane thin-walled curved box beam under the action of in-plane loading [5, 6]. Zhang et al. proposed a new finite element method which is considering eight cross-sectional deformation modes [7–9]. This requires the introduction of a section feature deformation described by a high-order function, that is, a high-order feature deformation. The strain values of the deformed configuration were calculated in terms of the displacement values and the initial curvature by Afnani et al. [10]. Fazlali et al. presented an analytical solution for the elastic-plastic pure bending of a linear kinematic-hardening curved beam with a rectangular cross-section [11].

However, previous literature has made assumptions regarding deformation theory, the rigidity of the section, and the effect of curvature, in the above articles on thin-walled curved beams, whether in finite-element analysis, linear viscoelastic analysis [12], one-dimensional high-order theory [13], or cross-section deformation mode analysis [14, 15]. In theoretical analysis, the analysis is always predictive, regardless of its accuracy. Thus, current analysis lack analytical methods based on actual experimental data.

As to the finite element analysis factors of plane problems on beam structures, accuracy and convergency are the most important issues among them. In one of the most recent works in the fields of assumed strain elements, Rezaiee-Pajand published some numerical articles [16–21] related to high-order strains, which expanded the authors' general view of numerical analysis.

Some researchers studied the surrogate of FEM by deep learning, which mainly trains the deep neural networks from datasets obtained from FEM. Hongwei Guo et al. [22] proposed a deep collocation method (DCM) for thin plate bending problems, which predicted maximum transverse with increasing layers is studied in order to show the convergence of deep collocation method in solving the plate bending problem. E. Samaniego et al. [23] explore the possibility of using a Deep Neural Network- (DNN-) based solver for Partial Differential Equations (PDEs). Solving them is a crucial step towards a precise knowledge of the behavior of natural and engineered systems.

To enable shape sensing analyses of beam structures undergoing bending deformation, Marco Gherlone et al. demonstrated that the recently presented iFEM for beam is reliable when experimentally measured strains are used as input data [24, 25]. Adnan Kefal developed a new eight-node curved inverse-shell (iCS8) element based on iFEM methodology, in which the high accuracy and practical utility of the iCS8 element are demonstrated for different cylindrical marine structures through examining coarse iCS8 discretizations with dense and sparse sensor deployments [26–29].

In this paper, we present experimental and theoretical analysis methods for the static analysis of a thin-walled curved rectangular box beam under in-plane bending based on 11 feature deformation modes. The experimental method for measuring the actual deformation of each section in a curved beam structure is proposed, using a one-dimensional deformation experimental test theory. Numerical investigations is explored the convergence and accuracy analysis by normal finite element analysis, higher-order assumed strain plane element, deep collocation method element, and inverse finite element method, respectively. The out-of-plane and in-plane characteristic deformation modes derived in the formula are superimposed by transforming the axial, tangential, and normal deformation values into tensile and compression components. The one-dimensional deformation experimental test theory is first proposed, formulating the specific contributions of various deformation modes. In this way, the magnitude and trend of the influence of each low-order deformation, distortion, and warping mode on the actual deformation are determined, and the significance of distortion and warping in actual curved beams subjected to in-plane loads is verified. This theory can be used in cases where it is difficult to measure and apply the measurement of the deformation distribution of various elastomer materials and structures to better understand the mechanical properties of the structure. The actual influence of distortion and warping in high-order deformation on deformation is reflected by actual data and a predictive curve. The low-order and high-order deformation modes of the box curved beam subjected to an in-plane load are thus obtained.

## 2. Theoretical Analysis

**Figure 1:**
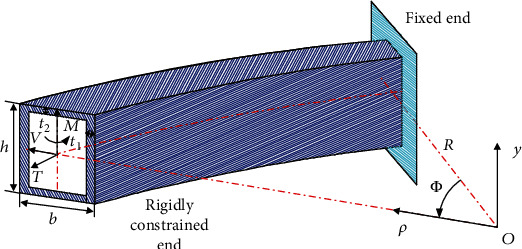
Geometry and coordinate system for cantilevered thin-walled curved box beam subjected to external loads.

The geometrical dimensions of the thin-walled curved box beam shown in Figure1 include *ρ*, *Φ*, and *y*, where *ρ* is the radial coordinate, *Φ* is the angular coordinate starting from the fixed end, and *y* is the ordinate. The height and width of the thin-walled curved beam structure are *h* and *b*, respectively, and the thicknesses of the wall are *t*_1_ and *t*_2_, respectively. The cantilever beam structure of the thin-walled curved beam mechanism is fixed at one end, and the load is applied to the other free end. The load in the plane comprises the tangential force *T*, radial force *V*, and in-plane bending moment *M*.

In order to determine the contour displacement field, the cross-section analysis of the thin-walled curved rectangular box beam is utilized. Firstly, research on all deformations of the predecessors is performed, and the high-order deformation is extracted by removing the low-order deformation components from the deformation [9]. A feature deformation is identified, and its shape function is defined to eliminate the influence of that feature deformation in the next function mode. The shape function of the section deformation and the wall envelope area are iterated. Eleven feature deformations are identified, in which the deformations 1st, 2nd, 3rd, 7th, 8th, and 9th are low-order deformation modes; the 4th, 5th, and 6th are high-order deformation modes of in-plane distortion, and 10th and 11th are high-order warping deformation modes.

According to the Timoshenko theory, the six low-order deformations include the translational displacement of the section by torsion, axial expansion, and rotational displacement about the *x*- and the *y*-axis. Modes 4th-6th are thus torsional distortion, uniaxial symmetric bending distortion, and biaxial symmetric bending distortion; modes 10th and 11th are torsional warping and bending warping, respectively, in the out-of-plane high-order deformation.

**Figure 2:**
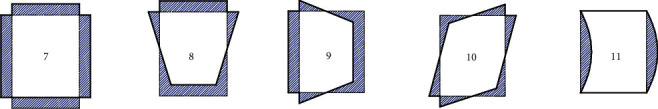
Cross-sectional deformation modes from out-of-plane into in-plane.

The actual deformation of the cross-section of all thin-walled curved rectangular box beams is the superposition of vectors of the above low- and high-order deformations [5]. The low- and high-order deformations are composed of axial, tangential, and normal components. By superimposing the out-of-plane and in-plane deformations, this study experimentally determines the actual deformation of the section via only tension and compression components. The axial deformation of the wall is perpendicular to the cross-section, with the negative value perpendicular to downward face, meaning that tension is positive. As for the normal and tangential deformation, the outer contour of the siding is positive, and the inside of the contour is negative. The deformation vectors of the above three directions are extracted into scalar deformation types in a unified plane by tension and compression. The out-of-plane deformation by modes 7th-11th is thus transformed to an in-plane scalar form, as shown in Figure2, which can be superimposed with the deformation vectors 1st-6th to determine the actual deformation of any point on the wall.

**Figure 3:**
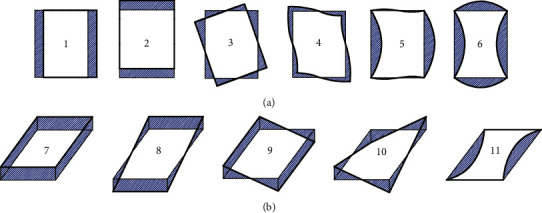
Cross-sectional deformation modes of a thin-walled rectangular beam.

In order to obtain the in-plane section deformation function of the thin-walled curved box beam, it is necessary to consider the eleven types of section deformation modes listed in the previous section (as shown in Figure 3). We consider the four walls of the rectangular section of the box beam are as the research objects, to accurately calculate the expressions of various deformed shape functions. As shown in Figure1, the thin-walled curved box beam is subjected to in-plane radial loading. The deformation is not symmetrical about *y* because of the curvature. This deformation can be decomposed into symmetric, asymmetric, and high-order modes, namely, low-order and high-order modes.

**Figure 4:**
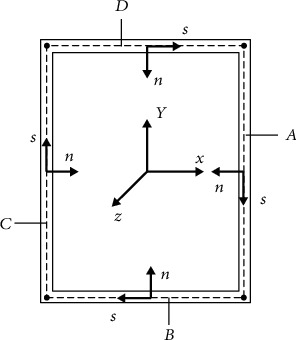
Local system of wall.

A local coordinate system of the wall, as shown in Figure4, is established, in which *n* and *s* represent the normal and tangential directions, respectively.

The deformation expression *χ*_b_(*ϕ*) of the contour displacement component of the rectangular section of the curved beam [7] can be expressed as

(1) $$\begin{array}{c} u_{nj}\left(s_{j},\phi \right)=\psi_{nj}^{\chi_{b}}\left(s_{j}\right)\chi_{b}\left(\phi \right), \end{array}$$

(2) $$\begin{array}{c} u_{sj}\left(s_{j},\phi \right)=\psi_{sj}^{\chi_{b}}\left(s_{j}\right)\chi_{b}\left(\phi \right), \end{array}$$

(3) $$\begin{array}{c} u_{\phi j}\left(s_{j},\phi \right)=\psi_{\phi j}^{\chi_{b}}\left(s_{j}\right)\chi_{b}\left(\phi \right). \end{array}$$

*ψ*_*rj*_^*χ*_*b*_^(*s*_*j*_) represents the shape function of the wall of the beam and is used to describe the shape of the line in the profile of the section; *j* indicates the *j*th wall, *r* is the direction of the deformation in the section wall, such as  *r* = *n*, *s*, *φ*, respectively, for the normal, tangential, and radial directions. *χ*_*b*_ denotes the generalized displacement of a point on the midline of the section, which is used to describe the amplitude variation of the in-plane and out-of-plane feature deformations along the axis.

The load of the analyzed curved beam is the in-plane bending load, while ignoring the unloading effect and shear lag. An approximate shape function must be derived using a one-quarter or one-half model of the entire section model. Because both the geometry and the external load are curved by the inner plane, the curvature is symmetric about the two axes of the hollow rectangular section.

Three orthogonal displacement components are used to represent the displacement of the point (*α*, *s*) on the midline of the section at time *t*: the axial component ${\tilde{u}}_{r}$, the tangential component ${\tilde{v}}_{r}$, and the normal component ${\tilde{w}}_{r}$, in which the three displacement components are along the coordinate axis (*α*, *s*, *n*) direction is positive. The three displacement components can be expressed in the form of the sum of finite terms by modal superposition method, namely,

(4) $$\begin{array}{c} {\tilde{u}}_{r}\left(\alpha ,s;t\right)=\overset{11}{\underset{i=1}{\sum }}\chi_{ri}\left(\alpha ;t\right)\varphi_{ri}\left(s\right), \end{array}$$

(5) $$\begin{array}{c} {\tilde{v}}_{r}\left(\alpha ,s;t\right)=\overset{11}{\underset{i=1}{\sum }}\chi_{ri}\left(\alpha ;t\right)\psi_{ri}\left(s\right), \end{array}$$

(6) $$\begin{array}{c} {\tilde{w}}_{r}\left(\alpha ,s;t\right)=\overset{11}{\underset{i=1}{\sum }}\chi_{ri}\left(\alpha ;t\right)\phi_{ri}\left(s\right), \end{array}$$

where *χ*_*ri*_(*α*; *t*) represents the generalized displacement of a point (*α*, *s*) on the midline of the section at time *t*, which is used to describe the amplitude change of the in-plane and out-of-plane characteristic deformation along the axis; while *φ*_*ri*_(*s*), *ψ*_*ri*_(*s*), and *ϕ*_*ri*_(*s*) also known as generalized coordinates, it is used to describe the shape change of the section along the center line of the section profile. Considering the influence of the axial and tangential displacement due to the deflection of the wall, the three-dimensional displacement component vector is obtained from the two-dimensional displacement component vector expressed as

(7) $$\begin{array}{c} {\text{x}}_{r}={\text{H}}_{r}{\text{X}}_{r}, \end{array}$$

where 2D displacement component vector X_*r*_ obtained by Equation(7), 3D *d*isplacement component vector x_*r*_ and conversion matrix *H*_*r*_ expressed as

(8) $$\begin{array}{c} {\text{x}}_{r}={\left[u_{r}\left(\alpha ,s,n;t\right)v_{r}\left(\alpha ,s,n;t\right)v_{r}\left(\alpha ,s,n;t\right)w_{r}\left(\alpha ,s,n;t\right)\right]}^{T}, \end{array}$$

(9) $$\begin{array}{c} H_{r}={\left[\begin{array}{ccc} 1 & 0 & -n\frac{\partial }{\rho \partial \alpha }\\ 0 & 1 & -n\frac{\partial }{\partial s}\\ 0 & 0 & 1 \end{array}\right]}^{T}, \end{array}$$

(10) $$\begin{array}{c} X_{r}={\left[\begin{array}{ccc} {\tilde{u}}_{r}\left(\alpha ,s,n;t\right) & {\tilde{v}}_{r}\left(\alpha ,s,n;t\right) & {\tilde{w}}_{r}\left(\alpha ,s,n;t\right) \end{array}\right]}^{T}. \end{array}$$

Kirchhoff's hypothesis on bending problem is adopted for the strain field and stress field: straight line hypothesis, namely, the thickness is constant; the normal stress on the midplane is much smaller than the other stress component hypothesis, and the midplane has no expansion and contraction assumption. At the same time, using linear elastic constitutive relationship expressed as

(11) $$\begin{array}{c} \sigma_{r}=E_{r}\varepsilon_{r}, \end{array}$$

where *σ*_*r*_ represents stress component column vector, *ε*_*r*_ represents strain component column vector, which could be obtained easily according to the geometric equations and matrix expressions, while it should be noted the constitutive matrix *E*_*r*_ in the plane stress is expressed as

(12) $$\begin{array}{c} E_{r}=\left[\begin{array}{ccc} E_{1} & 0 & E_{1}\nu \\ 0 & G & 0\\ E_{1}\nu & 0 & E_{1} \end{array}\right], \end{array}$$

(13) $$\begin{array}{c} E_{1}=\frac{E}{1-\nu^{2}}, \end{array}$$

(14) $$\begin{array}{c} G=\frac{E}{2\left(1+\nu \right)}, \end{array}$$

where *E*, and *G* represent the Young's modulus and shear modulus of the material, respectively; *ν* represents the Poisson constant.

The kinematic equation of the curved beam segment is established by the energy method, it is necessary to determine the strain energy, external force potential energy, and kinetic energy of the structure; the strain energy of a curved beam is express as

(15) $$\begin{array}{c} U=\frac{1}{2}\int \int {\varepsilon_{r}}^{T}\sigma_{r}dAdz, \end{array}$$

where the two integrations are performed on the integration space of the beam axis direction and the cross-sectional area, respectively, regardless of nonlinear factors; the external force potential energy of the beam is express as

(16) $$\begin{array}{c} U_{P}=-\int \int {{\text{x}}_{r}}^{T}{\text{p}}_{r}dAdz-\int \int {\left({{\text{x}}_{r}}^{T}{\text{q}}_{r}d\Omega \right)}_{n=-t_{r}/2}dz-\int {\left[{{\text{x}}_{r}}^{T}{\bar{\sigma }}_{r}\right]}_{z=z_{1}}^{z=z_{2}}dA, \end{array}$$

where *z*_1_ and *z*_2_ represent axial coordinates at both ends of the beam (*z*_1_ < *z*_2_); *Ω* represents integral space of the s coordinate along the midline of the section; *p* is the force sequence vector of the body distribution acting on the middle surface; and *q* is the force sequence vector of the surface distribution acting on the beam surface; ${\bar{\sigma }}_{r}$ is the vector of force series acting on the cross section of the beam. In which the first integral range refers to the integral space of the beam axis direction and the cross-sectional area, the second integral range refers to the integral space of the cross-sectional area and the integral space of the *s*-coordinate along the center line of the section, and the third integral range refers to the direction of the beam axis. The kinetic energy is expressed as

(17) $$\begin{array}{c} T=\frac{1}{2}\int \int \frac{\partial {{\text{u}}_{r}}^{T}}{\partial t}{\Psi_{r}}^{T}{H_{r}}^{T}\eta H_{r}\Psi_{r}\frac{\partial {\text{u}}_{r}}{\partial t}dAdz. \end{array}$$

The Hamilton principle and Lagrange function are used to derive the kinematic differential equation of the curved beam. The Hamilton principle can be expressed as

(18) $$\begin{array}{c} \delta H=\delta \int_{t_{1}}^{t_{2}}Ldt, \end{array}$$

where *H* represents Hamilton and *L* represents Lagrange function.

(19) $$\begin{array}{c} L=T-U-U_{P}. \end{array}$$

## 3. Numerical Investigations

### 3.1. Finite Element Analysis

**Figure 5:**
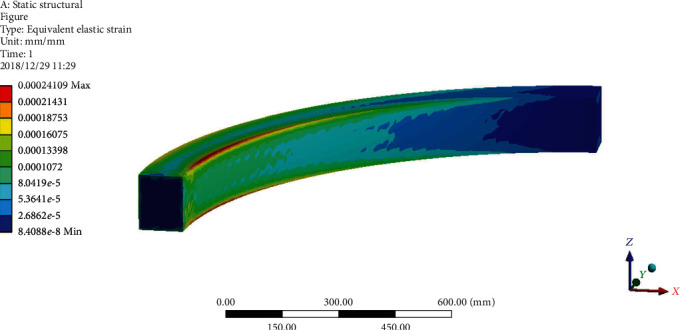
Finite element analysis of curved beam structure under load.

A full model representation includes supports and loading, in which the top end of the beam are totally restrained at the position of *φ* = 0°, acting as fixed supports, and a point load is applied at the free end of the beam; this load will create a moment under total loads of 300 N and 1000 N and the forces applied at the position of *φ* = 90° along the *y*-axis. Analysis is carried out by linearly increasing the load from zero to a maximum value given by the expected total capacity of the structure, whose model is meshed into thin shell elements having the material parameters of *E* = 200 MPa. Finite model is meshed into 809 elements and 2046 nodes with thin shell element, which the results obtained under the load of 1000 N in shown in Figure5.

Generally speaking, the field variable selection is the most important step in finite element technology. Obviously, the accuracy of this approximation depends on the interpolation function assigned to the element. Different types of mathematical functions can be used to express these functions. However, one of the most stable forms is a polynomial basis of degree *𝒫*:

(20) $$\begin{array}{c} P^{P}=\left[\begin{array}{ccc} x & y & \begin{array}{ccc} x^{2} & xy & \begin{array}{ccc} y^{2} & \cdots & y^{P} \end{array} \end{array} \end{array}\right]\tilde{a}, \end{array}$$

(21) $$\begin{array}{c} \tilde{a}={\left[a_{i1}a_{i2}\cdots \right]}^{T}. \end{array}$$

The approximation field consists of two main parts: (a) interpolation function—the order of this function determines the accuracy and speed of convergence to achieve a precise response. The degree of convergence and sustainability gradient depends on the degree of *𝒫*. (b) Additional carrier—according to the formula method, its definition is different. In some formulas based on displacement, it is called a degree of freedom vector. In the displacement field method, these parameters are obtained by various methods, but in the natural assumed strain method, these strain states are based on the application of a series of optimal criteria to reduce the stress, according to the degree of freedom and derived from the distribution of geometric shapes. The number of degrees of freedom required. The most commonly used best criteria are balance and compatibility equations. In addition to forcing a minimum energy level to the finite element, applying balance can also increase the convergence speed of the resulting element.

### 3.2. Higher-Order Assumed Strain Plane Element

The traditional plane element analysis needs to be divided into a very fine mesh to get accurate results, and the convergence speed is too slow. In order to improve the convergence speed and accuracy of finite element analysis for planar elements, in this paper, we adopted a new robust membrane finite element for the analysis of plane problems proposed by Rezaiee-Pajand, which has triangular geometry with four nodes and 11 degrees of freedom for the element, in which each of the three vertex nodes has three degrees of freedom, two displacements, and one drilling. The fourth node that is located inside the element has only two translational degrees of freedom. Three different meshes are used to analyze this structure, namely, 1 × 6, 2 × 12, and 4 × 24. These mesh styles are named based on the number of quadrilateral elements used in them. Of note, to analyze using triangular elements, each quadrilateral element is divided into two triangular elements.

**Figure 6:**
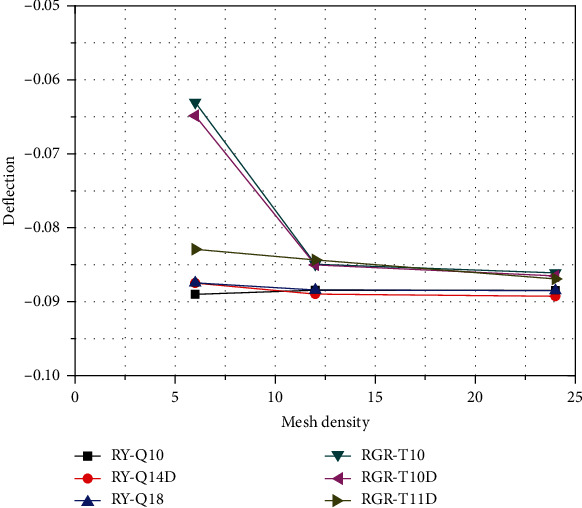
Convergence of the normalized tip deflection of the thin curved beam.

In order to compare the convergence rate and accuracy of various element types, tip deflection of the curved beam under applied load was computed, which proposed element in the analysis of curved structures; a thin curved cantilever beam loaded by a transverse load at its free end is analyzed. The beam is made of the elastic material with a modulus of elasticity and Poisson's ratio equal to 10^7^ and 0.25, respectively, and its inner radius and thickness are 4.12 and 0.1 unit. The beam is analyzed by three different mesh and the results of vertical displacement of the structural tip are listed in Table 1. The near-exact value is reported by Choo et al. (2006) is equal to 0.08734 [30]. The convergence curves for different elements are depicted in Figure6.

As shown in Table 1, the tip deflection values of element analysis with different mesh densities from 1 × 6 to 2 × 12 and 4 × 24 can be seen that the convergence of the triangular elements is significant; in the triangular element, the convergence speed and accuracy of the triangular element with 4 nodes and 10 degrees of freedom are better than the elements with 5 nodes and 10 degrees of freedom and 7 nodes and 10 degrees of freedom, besides the element with 6 nodes and 10 degrees of freedom (all translational degrees of freedom, no rotational degrees of freedom) excluded for high order analysis.

**Figure 7:**
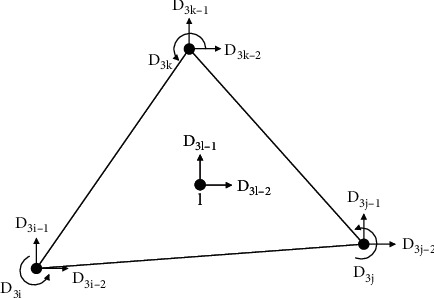
Four-node triangular element with incomplete second-order strain field.

Even the obtained results show that triangular plane elements with four-node and 11 degree of freedoms [31] perform better than the other elements shown in Figure7. All of the above, as to finite plane element analysis of the curved beam structures, the triangular element with 4 nodes and 11 degrees of freedom has fast convergence speed and high analysis accuracy.

### 3.3. Deep Collocation Method

This deep collocation method can be seen as a truly mesh-free method without the need of background grids.

This section introduces the deep collocation method used to solve the Kirchhoff board bending problem. This method is a widely used method to find the numerical solutions of normal, partial differential, and integral equations. Among the control theories, it is a popular method for trajectory optimization. Usually, a set of randomly distributed points (also called collocation points) is used to represent the required trajectory, which minimizes the loss function while satisfying a set of constraints. The collocation method is often relatively insensitive to instability and is a feasible method for training deep neural networks.

First, the collocation point is expressed as a discretized physical domain. Another set of collocation points is used to discretize the boundary conditions. Then, the feed forward deep neural network [23] is used to approximate the lateral deflection *w*. Therefore, a loss function can be constructed to find optimal hyperparameters by approximating the boundary conditions to the minimization of the governing equations. The purpose of this section is to seek a series of approximate deflection parameters to minimize the loss function (*θ*). If the function value is small, the approximate deflection value is very close to the value that satisfies the driving equation. The thin plate bending problem solved by the deep collocation method can be simplified into an optimization problem. In the deep learning Tensorflow framework, various optimizers are used. One of the most widely used optimization methods is the Adam optimization algorithm, which is also used in the numerical research of this article. The idea is to decrease at the collocation point *x*_*i*_, using the Adam-based learning rate *α*_*i*_, and then the process in Equation (22) is repeated until a convergence criterion is satisfied.

(22) $$\begin{array}{c} \theta_{i+1}=\theta_{i}+\alpha_{i}\nabla_{\theta }L\left(x_{i};\theta \right), \end{array}$$

where ∇ is commonly called biharmonic operator.

**Figure 8:**
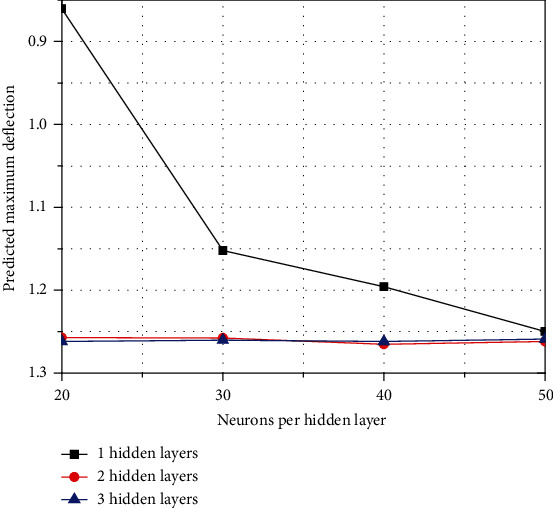
The relative deflection with varying hidden layers and neurons.

For the clamped load situation, a deep feedforward neural network with increased layers and neurons was studied to verify the convergence of the scheme. First, the maximum center deflection is shown in Table 2, which is calculated for the number of different layers and neurons and compared with Timoshenko's exact solution shown in Figure8. The result of deep collection is the most consistent with the exact solution. However, for a neural network with a single hidden layer, even with 60 neurons, the result is not very accurate. As the number of neurons increases, the results are indeed more accurate for neural networks with a single hidden layer. This can be observed for the other two hidden layer types. In addition, as the number of hidden layers increases, the result is much more accurate than that of a single hidden layer neural network.

### 3.4. Inverse Finite Element Method

The Timoshenko beam theory is adopted, and the discretization using the C0 continuous inverse element is adopted through the variational principle. The three-dimensional displacement field of the beam structure is reconstructed under the condition of ensuring the least squares compatibility between the measured strain and the strain interpolated by the inverse element. Then, describe the experimental setup. Thin-walled cantilever beams bear different static and dynamic loads. First, the measured surface strain is used as input data for shape sensing through a single inverse element. For the same test case, more and more inverse elements are also used to study convergence. Then, compare the deflection recovered by iFEM with the deflection measured experimentally. For static loads, the accuracy and convergence of iFEM measurement errors are proved.

As an example to illustrate the analysis of convergence and accuracy under the shape sensing of the inverse finite element method. Under the condition of tip force in static testing, one end is fully constrained, and the free end is loaded. The analysis of the working conditions under the load condition evaluates the accuracy of the solution by predicting the percentage difference of the displacement and the rotation relative to the experimentally measured rotation. The percentage difference is defined as the relative error between the predicted value of the end deflection and the actual measured value. It mainly analyzes the change value caused by the number of inverse elements. For all load conditions and using any strain gauge configuration considered, the end deflection predicted by iFEM. The difference with the measured value meets the error requirement range.

**Figure 9:**
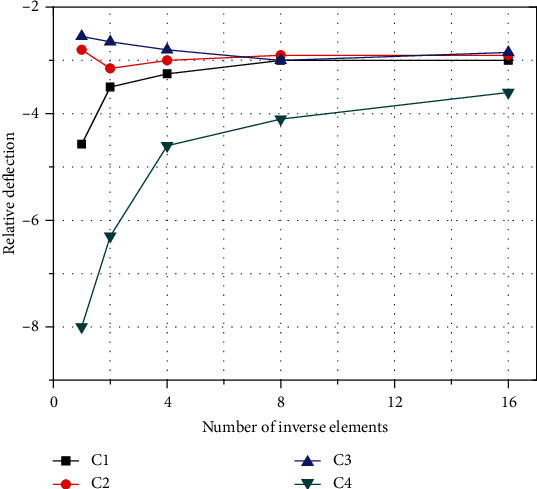
Relative tip deflection in iFEM-predicted of different strain gauges arrangement. C1: six strain gauges in one axial location; C2: six strain gauges in two axial locations; C3: eight strain gauges in two axial locations; C4: eight strain gauges in three axial locations.

As shown in Figure 9, more and more inverse elements obtained the end displacement results under load conditions. All elements are distributed with strain gauges corresponding to each curve. As the number of antielements increases, the percentage difference in the predicted iFEM deflection will converge to a value less than 6%. Each input strain is obtained by linear fitting to three different values. When using fitted strain data, the main advantage of applying the fitting process to the original data is that a large number of inverse elements can be used to discretize the frame members without the need to obtain additional data. In this case, it is advantageous to use higher fidelity discretization for the iFEM model. Generally, the number of antielements to be used is related to the complexity of the applied load and the expected structural deformation. Using the fitted data, the results obtained by using different strain gauge configurations will converge to almost the same value. At the same time, the more strain gauges, the higher the convergence efficiency. At the same time, the strain gauges are distributed at the most, with the same amount of strain. The more the instrument is distributed, the faster the convergence efficiency. Therefore, as the iFEM algorithm, in discretization applications, more strain gauges should be used, and they should be evenly distributed in each position of the structure, which will help the convergence speed and achieve the accurate value faster.

## 4. Experimental Investigation

### 4.1. Test Specimens

The curved plates of the curved beam structure are formed by bending two flat plates to have a certain radius of curvature and then welding them with the upper and lower flat plates. At this point, the two curved plates are marked before bending. A butt-welded square box cross-sectional shape is used in the curved beams. Each wall plate is composed of Q235 (235 MPa) normal strength steel, and the steel plate with a thickness of 2.2 mm is flame-cut.

**Figure 10:**
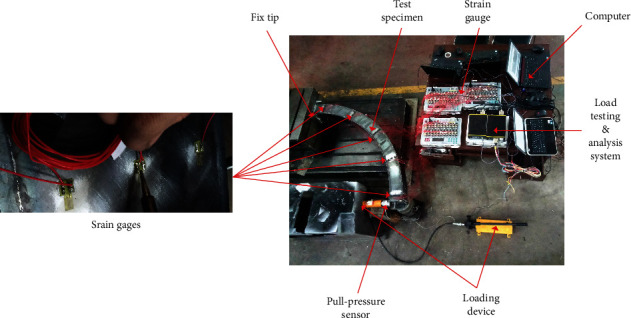
Typical test setup.

The beam is of width *b* = 120 mm, height *h* = 150 mm, wall thickness *t* = 2.2 mm, section radius of centerline *R* = 1 m, and central angle *φ* = 90°. The experimental sample structure is welded from four Q235-grade steel plates to form a thin-walled curved beam; it was stored at rest for half a year after welding. In order to prevent the influence of uniqueness on the data, four samples with the same materials and parameters were tested. To research the properties of a thin-walled curved box beam, the fixed end fully and rigidly constrained by a rigid plate. The in-plane load V is applied as a radial load of 1000 N, and the load direction is as shown in Figure10.

### 4.2. Specimen Labeling

**Figure 11:**
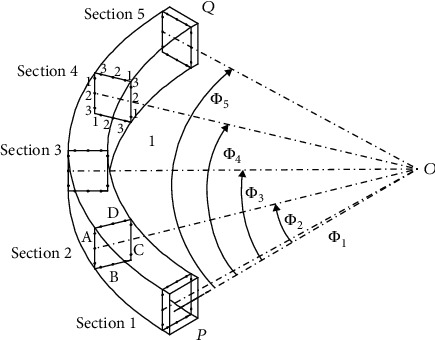
Test mark information in the curved beam.

The mark number of each detection point is *ijk* as shown in Figure 11, where *i* represents the section of the central axis of the curved beam section with the point *O* as the origin, and curve PQ is the central axis of the section of the thin-walled curved box beam *i* = 1, 2, 3, 4, and 5, with point *P* as the reference point, according to the angle *Φ*_*i*_ of *Φ*_1_ = 1.72°, *Φ*_2_ = 22.5°, *Φ*_3_ = 45°, *Φ*_4_ = 67.5°, and *Φ*_5_ = 88.28°, as shown in Figure 9, respectively. To ensure the test points are evenly distributed, the angle between the 1th and 5th sections is defined by the necessary distance of 20 mm to the end to avoid the welding position; point *P* of the section is the constraint position.

**Figure 12:**
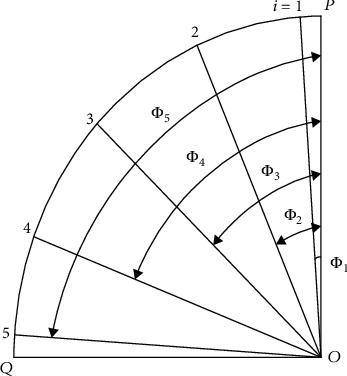
Test section distribution in curved beam.

Point *Q* represents the loading position, as shown in Figure12, *j* represents the *j*th wall of the ith section, which has four walls.

**Figure 13:**
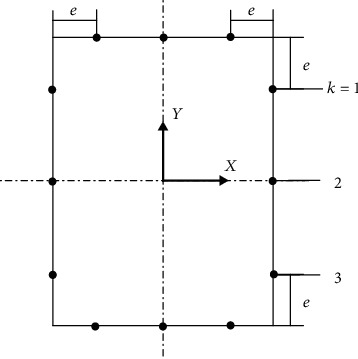
Test location markings on the cross-section.

*A* indicates the wall of the outer diameter, and the other wall plates are arranged in clockwise order, with wall plate *B* located below, *C* at the inner diameter, and *D* above, *k* represents the position of the axially arranged resistance strain gauge where the *j*th wall of the *i* section is located; each wall has three positions in every section, for a total of 12 positions in each section. *k* = 1 is the starting test point, the other test points are arranged clockwise, where the o'clock position relative to the edge of the wall is *e* = 20 mm in order to avoid the welding position of the end, point 2 is the center position of each wall, and points 3 and 1 are symmetric about point 2 (as shown in Figure 13).

Strain gauges were attached to each of the 60 positions of the five sections on each of the four curved beam samples, and the strain values at each position were tested by a DH3820 high-speed static strain analysis system.

### 4.3. Test Results

**Figure 14:**
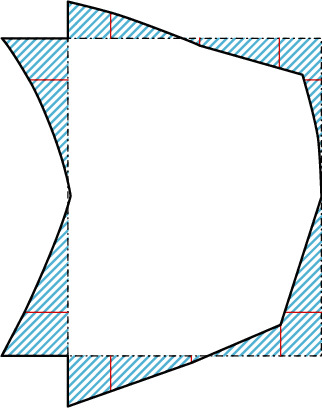
Sectional strain of each test point.

The four sets of experimental data are similar. The results shown in Figure14 show the strain value amplified by 2 × 10^6^, because the experimental strain values are otherwise too small to see. The deformation diagram of positions 1-5 in the cross-section are shown in the figure. The shapes are formed by the strain values at the 12 test locations on each section; the thick solid line is the deformed shape, while the double dotted line is the nondeformed shape. The thin red solid line represents the strain value used to test the deformation change of the *k* point for this position; the shape is determined by this parameter. Because the experimental process can only measure the tensile or compressive strain of the test point at the test point itself, it is impossible to measure the strain at a certain position by in-plane deformation or out-of-plane deformation.

**Figure 15:**
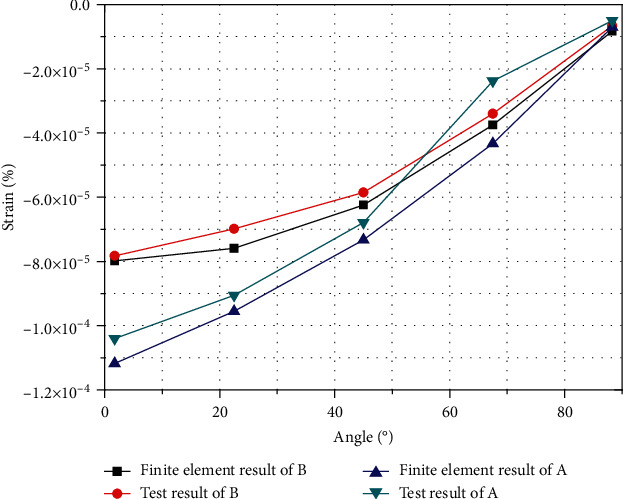
Comparison of finite element analysis and experimental results of strain at various positions on curved surface of curved beam.

Therefore, in the experiment, the nondeformed cross-sectional outline is set to a positive value as the tensile strain, and the negative value is the compressive strain inward, formed as shown in the figure. As shown in Figure15, compared with the experiment, the theoretical analysis has a small error, within the allowable range, and mutually verified its accuracy.

## 5. One-Dimensional Deformation Experimental Test Theory

The strain value of each position of each wall of each section measured by the experimental method is scalar and composed of the axial, normal, and tangential strains. Although the actual deformation on the theoretical level is formed by distortion and warping in the low- and high-order deformation, the influence of the various deformation forms on the actual deformation must be determined. This study proposes an experimental test theory, which first calculates the expressions of various deformation modes in the axial, normal, and tangential components and then uses the undetermined coefficient method to calculate the influence coefficient *χ*_*b*_(*ϕ*) of the various deformation modes. By substituting this coefficient into the formulas of low-order, distortion, and warping effects, the respective influences are obtained, and the distribution of the test points at each position of the curved beam is used to infer the distribution of the entire curved beam. It thus can be determined whether there is any shape deformation effect that can be ignored.

(23) $$\begin{array}{c} u_{T}\left(s_{j},\phi \right)=u_{L}\left(s_{j},\phi \right)+u_{D}\left(s_{j},\phi \right)+u_{W}\left(s_{j},\phi \right). \end{array}$$

The one-dimensional deformation effect formula for the thin-walled curved rectangular box beam is shown in Equation (23), where *u*_*L*_ is the low-order effect and *u*_*D*_ and *u*_*W*_ are the effects of distortion and warping, respectively.

The low-order effect indicates six kinds of deformation mode in Equation (16), where *ψ*_*L*_^*χ*_*b*_^ is a low-order shape function matrix concluding the six basic low-order deformation modes, while *χ*_*b*_^*L*^ is a low-order influence coefficient matrix. Equation (17) illustrates the distortion effect, where *ψ*_*D*_^*χ*_*b*_^ and *χ*_*b*_^*D*^ represent the distortion effect shape function matrix and the distortion influence coefficient matrix, respectively, and the distortion types include bending and torsional distortion.

For the warping influence equation, *ψ*_*W*_^*χ*_*b*_^and *χ*_*b*_^*W*^ represent the warping function matrix and warping influence coefficients matrix, respectively, and the warping forms include two types of bending and torsion warping as shown in Equation(18).

Low-order effect:

(24) $$\begin{array}{c} u_{L}\left(s_{j},\phi \right)=\psi_{L}^{\chi_{b}}\left(s\right)\chi_{b}^{L}, \end{array}$$

(25) $$\begin{array}{c} \chi_{b}^{L}=\left[\chi_{b1}^{L},\chi_{b2}^{L},\chi_{b3}^{L},\chi_{b7}^{L},\chi_{b8}^{L},\chi_{b9}^{L}\right], \end{array}$$

(26) $$\begin{array}{c} \psi_{L}^{\chi_{b}}\left(s\right)={\left[\psi_{n1}^{\chi_{b}}\left(s\right),\psi_{n2}^{\chi_{b}}\left(s\right),\psi_{n3}^{\chi_{b}}\left(s\right),\psi_{\varphi 7}^{\chi_{b}}\left(s\right),\psi_{\varphi 8}^{\chi_{b}}\left(s\right),\psi_{\varphi 9}^{\chi_{b}}\left(s\right)\right]}^{T}. \end{array}$$

Distortion effect:

(27) $$\begin{array}{c} u_{D}\left(s_{j},\phi \right)=\psi_{D}^{\chi_{b}}\left(s\right)\chi_{b}^{D}, \end{array}$$

(28) $$\begin{array}{c} \chi_{b}^{D}=\left[\chi_{b5}^{D},\chi_{b6}^{D}\right], \end{array}$$

(29) $$\begin{array}{c} \psi_{D}^{\chi_{b}}\left(s\right)={\left[\psi_{n5}^{\chi_{b}}\left(s\right),\psi_{n6}^{\chi_{b}}\left(s\right)\right]}^{T}. \end{array}$$

Warping effect:

(30) $$\begin{array}{c} u_{W}\left(s_{j},\phi \right)=\psi_{W}^{\chi_{b}}\left(s\right)\chi_{b}^{W}, \end{array}$$

(31) $$\begin{array}{c} \chi_{b}^{W}=\left[\chi_{b10}^{W},\chi_{b11}^{W}\right], \end{array}$$

(32) $$\begin{array}{c} \psi_{W}^{\chi_{b}}\left(s\right)={\left[\psi_{n10}^{\chi_{b}}\left(s\right),\psi_{n11}^{\chi_{b}}\left(s\right)\right]}^{T}. \end{array}$$

This theory can be used in cases where it is difficult to measure the feature deformation modes, and it is impossible to distinguish between the out-of-plane and in-plane deformation modes. At the same time, the theory can be applied to the measurement of the deformation distribution of various elastomer materials and structures, to better understand the mechanical properties of the structure.

### 5.1. Derivation of Deformation Function Expression

#### 5.1.1. Derivation of Low-Order Shape Function Expression

The deformation of the lower-order features can cause the main deformation of the section, which is the main deformation feature. The six low-order deformations include translational displacement by torsion, axial expansion, and rotation of the section about the *x*-axis and the *y*-axis, respectively. The characteristic deformations 1st, 2nd, and 3rd are normal deformations, and the characteristic deformations 7th, 8th, and 9th are axial deformations. The above deformation modes are, respectively, used as the main forms of low-order deformation, and the expression [1] is given by

(33) $$\begin{array}{c} \psi_{n1}^{\chi_{b}}\left(s\right)=\left\{\begin{array}{cc} -1, & s\in \left[s_{AD},s_{AB}\right),\\ 0, & s\in \left[s_{AB},s_{BC}\right),\\ 1, & s\in \left[s_{BC},s_{CD}\right),\\ 0, & s\in \left[s_{CD},s_{DA}\right), \end{array}\right. \end{array}$$

(34) $$\begin{array}{c} \psi_{n2}^{\chi_{b}}\left(s\right)=\left\{\begin{array}{cc} 0, & s\in \left[s_{AD},s_{AB}\right),\\ 1, & s\in \left[s_{AB},s_{BC}\right),\\ 0, & s\in \left[s_{BC},s_{CD}\right),\\ -1, & s\in \left[s_{CD},s_{DA}\right), \end{array}\right. \end{array}$$

(35) $$\begin{array}{c} \psi_{n3}^{\chi_{b}}\left(s\right)\left\{\begin{array}{c} -\left(s-s_{AD}\right)+\frac{h}{2},s\in \left[\left.s_{AD},s_{AB}\right),\right.\\ -\left(s-s_{AB}\right)+\frac{b}{2},s\in \left[\left.s_{AB},s_{BC}\right)\right.,\\ -\left(s-s_{BC}\right)+\frac{h}{2},s\in \left[\left.s_{BC},s_{CD}\right),\right.\\ -\left(s-s_{CD}\right)+\frac{b}{2},s\in \left[\left.s_{CD},s_{DA}\right)\right., \end{array}\right. \end{array}$$

(36) $$\begin{array}{c} \psi_{\varphi 7}^{\chi_{b}}\left(s\right)=1, \end{array}$$

(37) $$\begin{array}{c} \psi_{\varphi 8}^{\chi_{b}}\left(s\right)=\left\{\begin{array}{cc} -\frac{2}{h}{\left(s-s_{AD}\right)}^{2}+1, & s\in \left[s_{AD},s_{AB}\right),\\ 1, & s\in \left[s_{AB},s_{BC}\right),\\ \frac{2}{h}{\left(s-s_{BC}\right)}^{2}+1, & s\in \left[s_{BC},s_{CD}\right),\\ -1, & s\in \left[s_{CD},s_{DA}\right), \end{array}\right. \end{array}$$

(38) $$\begin{array}{c} \psi_{\varphi 9}^{\chi_{b}}\left(s\right)=\left\{\begin{array}{cc} -1, & s\in \left[s_{AD},s_{AB}\right),\\ \frac{2}{b}\left(s-s_{AB}\right)-1, & s\in \left[s_{AB},s_{BC}\right),\\ 1, & s\in \left[s_{BC},s_{CD}\right),\\ -\frac{2}{b}\left(s-s_{CD}\right)+1, & s\in \left[s_{CD},s_{DA}\right). \end{array}\right. \end{array}$$

#### 5.1.2. Derivation of Distortion Shape Function Expression

**Figure 16:**
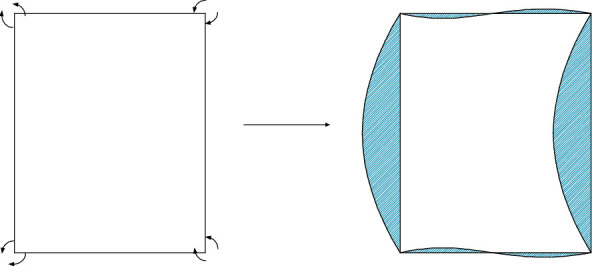
The bending moment of the wall on the section.

The high-order influence mainly comprises of the influences of distortion and warping, which is analyzed based on Zhang et al. [9]. The distortion is caused by the deflection of the section wall, and the bending moment on the wall is directly related. Because of the force of the structure of the cantilever beam, the direction and form of each wall plate can be inferred to infer the determine of the bending moment on the cross-section according to the identified distortion shape (as shown in Figure 16).

The cross-sectional dimension of the curved beam section is much larger (>5) than the wall thickness thus satisfying the applicable range of the Euler-Bernoulli beam theory. Therefore, the normal deformation function *ψ*_*sj*_^*χ*_*b*_^(*s*_*j*_) can be approximated as a cubic polynomial:

(39) $$\begin{array}{c} \psi_{nj}^{\chi_{b}}\left(s\right)=a_{j}^{3}s^{3}+a_{j}^{2}s^{2}+a_{j}^{1}s+a_{j}^{0}. \end{array}$$

The superscripts “+” and “-” indicate the approach directions of the tangential coordinates in Equation (39); *a*_*j*_^*i*^ (*i* = 0, 1, 2, 3, *j* = 5), where *i* denotes the power of the polynomial and *j* denotes the jth shape deformation. Because the force mode determines the curved rectangular shape, the distortion type is only a single axis-symmetrical distortion type, so *j* can only be equal to 5, and the Euler-Bernoulli beam theory is determined by combining the geometric symmetry of the section and the bending moment balance condition. The other basic assumptions that must be used include:

1. Right angles must be maintained at the junctions of adjacent walls
2. The lateral expansion and contraction of the wall section can be ignored
3. The displacement on the same wall plate can be continuously differentiated
4. The displacement of both sides of the same node is continuous

**Figure 17:**
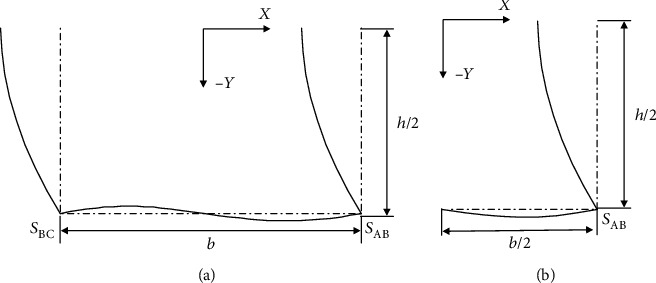
Rectangular section deformation under uniaxial symmetrical bending distortion. (a) Half-model. (b) Quarter-model.

According to the symmetry of the deformation and load on the *x*-axis, the distortion model of the section is simplified to the half-model shown in Figure17(a). The antisymmetric property about the *y*-axis is reused, and the section deformation model is further simplified to the quarter model shown in Figure 17(b).

Because the bending distortion is independent of the torsion, the deformation characteristics are shown in Figure10, and it is assumed that the wall is not telescopic, in order to consider that the section has no tangential displacement (*ψ*_*sj*_^*χ*_*b*_^(*s*) = 0). For the cubic polynomial describing the quarter-model deformation function, the eight unknown coefficients are determined according to the following conditions:

(1) According to the displacement continuity condition, the tangential displacement and the normal displacement at the intersection of the faces *A* and *B* must satisfy
  (40) $$\begin{array}{c} \psi_{n5}^{\chi_{b}}\left(s\right){\left|{}_{s_{\text{AB}}^{-}}=-\psi_{s5}^{\chi_{b}}\left(s\right)\right|}_{s_{\text{AB}}^{+}}, \end{array}$$
  (41) $$\begin{array}{c} \psi_{n5}^{\chi_{b}}\left(s\right){\left|{}_{s_{\text{AB}}^{+}}=-\psi_{s5}^{\chi_{b}}\left(s\right)\right|}_{s_{\text{AB}}^{-}}. \end{array}$$
(2) According to the bending moment balance condition at the corner joint, the deformation on both sides is opposite to that at the angular joint:
  (42) $$\begin{array}{c} t_{1}^{3}\frac{d^{2}\psi_{n5}^{\chi_{b}}\left(s\right)}{ds^{2}}{\left|{}_{s_{\text{AB}}^{-}}=t_{2}^{3}\frac{d^{2}\psi_{n5}^{\chi_{b}}\left(s\right)}{ds^{2}}\right|}_{s_{\text{AB}}^{+}}=0. \end{array}$$
(3) According to symmetric conditions about the *x*-axis:
  (43) $$\begin{array}{c} {\left.\frac{d\psi_{n5}^{\chi_{b}}\left(s\right)}{ds}\right|}_{s_{\text{AD}}+h/2}=0. \end{array}$$
(4) According to the antisymmetric conditions on the *y*-axis:
  (44) $$\begin{array}{c} {\left.\psi_{n5}^{\chi_{b}}\left(s\right)\right|}_{s_{\text{AB}}+b/2}=0. \end{array}$$
(5) According to the assumption that the corners are kept at right angles, the angles of the two side wall plates are equal
  (45) $$\begin{array}{c} \frac{d\psi_{n5}^{\chi_{b}}\left(s\right)}{ds}{\left|{}_{s_{\text{AB}}^{-}}=\frac{d\psi_{n5}^{\chi_{b}}\left(s\right)}{ds}\right|}_{s_{\text{AB}}^{+}}. \end{array}$$

According to the principle of normalization, the amplitude of middle point of wall A shown in Figure10 is 1:

(46) $$\begin{array}{c} {\left.\psi_{n5}^{\chi_{b}}\left(s\right)\right|}_{s_{\text{AD}}+h/2}=0. \end{array}$$

The eight linear independent equations in the simultaneous Equation(21), and the symmetric relationships yield the normal deformation function of the model:

(47) $$\begin{array}{c} \psi_{n5}^{\chi_{b}}\left(s\right)=\left\{\begin{array}{cc} \frac{4}{h^{3}}{\left(\frac{h}{2}-s+s_{AD}\right)}^{3}-\frac{6}{h^{2}}{\left(\frac{h}{2}-s+s_{AD}\right)}^{2}+1, & s\in \left[\left.s_{AD},s_{AD}+\frac{h}{2}\right),\right.\\ \frac{4}{h^{3}}{\left(s-s_{AD}-\frac{h}{2}\right)}^{3}-\frac{6}{h^{2}}{\left(s-s_{AD}-\frac{h}{2}\right)}^{2}-1, & s\in \left[s_{AD}+\frac{h}{2},s_{AB}\right),\\ \frac{12}{b^{2}h}{\left(s-s_{AB}\right)}^{3}-\frac{3}{h}\left(s-s_{AB}\right), & s\in \left[\left.s_{AB},s_{AB}+\frac{b}{2}\right),\right.\\ -\frac{12}{b^{2}h}{\left(b-s+s_{AB}\right)}^{3}+\frac{3}{h}\left(b-s+s_{AB}\right), & s\in \left[\left.s_{AB}+\frac{b}{2},s_{BC}\right),\right.\\ -\frac{4}{h^{3}}{\left(\frac{h}{2}-s+s_{BC}\right)}^{3}+\frac{6}{h^{2}}{\left(\frac{h}{2}-s+s_{BC}\right)}^{2}-1, & s\in \left[\left.s_{BC},s_{BC}+\frac{h}{2}\right),\right.\\ -\frac{4}{h^{3}}{\left(s-s_{BC}-\frac{h}{2}\right)}^{3}-\frac{6}{h^{2}}{\left(s-s_{BC}-\frac{h}{2}\right)}^{2}+1, & s\in \left[\left.s_{BC}+\frac{h}{2},s_{CD}\right),\right.\\ -\frac{12}{b^{2}h}{\left(s-s_{CD}\right)}^{3}+\frac{3}{h}\left(s-s_{CD}\right), & s\in \left[\left.s_{CD},s_{CD}+\frac{b}{2}\right),\right.\\ \frac{12}{b^{2}h}{\left(b-s+s_{CD}\right)}^{3}-\frac{3}{h}\left(b-s+s_{CD}\right), & s\in \left[\left.s_{CD}+\frac{b}{2},s_{AD}\right).\right. \end{array}\right. \end{array}$$

Because the axial deformations are orthogonal to each other, the axial deformation becomes 0:

(48) $$\begin{array}{c} \psi_{\varphi 5}^{\chi_{b}}\left(s\right)=0. \end{array}$$

According to the rule of strain measurement, the above deformation is not axially deformed, that is:

(49) $$\begin{array}{c} \psi_{5}^{\chi_{b}}\left(s\right)=\psi_{n5}^{\chi_{b}}\left(s\right). \end{array}$$

#### 5.1.3. Derivation of Warping Shape Function Expression

Warping is caused by uneven expansion of the siding. Based on the identified deformation characteristics of the characteristic deformations 10 and 11, the function is approximated by a quadratic polynomial:

(50) $$\begin{array}{c} \psi_{nj}^{\chi_{b}}\left(s\right)=a_{j}^{2}s^{2}+a_{j}^{1}s+a_{j}^{0}. \end{array}$$

**Figure 18:**
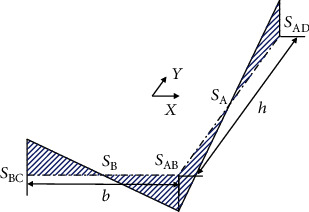
Torsion warping section deformation.

The coefficients in the formula are consistent with those mentioned in the previous section and are determined by the section deformation characteristics and geometric symmetry characteristics. According to the central symmetry characteristic of the torsional warping deformation, the biaxial symmetry characteristic of the cross-section can be simplified to solve the torsion warping model of the section to the shape function of the half-model (shown in Figure 18).

For the half-model, the next six conditions are used as the boundary conditions for determining the plane profile deformation function of the two side wall plates of the corner node:

1. Because the deformations on walls *A* and *B* are, respectively, opposed to *S*_*A*_and *S*_*B*_, the axial shape at the symmetry point becomes 0, that is,

(51) $$\begin{array}{c} \psi_{n10}^{\chi_{b}}\left(s\right){\left|{}_{s_{AD}+h/2}=\psi_{n10}^{\chi_{b}}\left(s\right)\right|}_{s_{AB}+b/2}=0. \end{array}$$

(2) The slopes at the two points of the central symmetry are equal, and the displacements are opposite. Taking the two end points of the wall plate as an example, joint continuity is assumed:

(52) $$\begin{array}{c} \frac{d\psi_{n10}^{\chi_{b}}\left(s\right)}{ds}{\left|{}_{s_{AD}^{+}}=\frac{d\psi_{n10}^{\chi_{b}}\left(s\right)}{ds}\right|}_{s_{AB}^{-}}, \end{array}$$

(53) $$\begin{array}{c} \frac{d\psi_{n10}^{\chi_{b}}\left(s\right)}{ds}{\left|{}_{s_{AB}^{+}}=\frac{d\psi_{n10}^{\chi_{b}}\left(s\right)}{ds}\right|}_{s_{BC}^{-}}, \end{array}$$

(54) $$\begin{array}{c} \psi_{n10}^{\chi_{b}}\left(s\right){\left|{}_{s_{AD}}=-\psi_{n10}^{\chi_{b}}\left(s\right)\right|}_{s_{AB}}, \end{array}$$

(55) $$\begin{array}{c} \psi_{n10}^{\chi_{b}}\left(s\right){\left|{}_{s_{AB}}=-\psi_{n10}^{\chi_{b}}\left(s\right)\right|}_{s_{BC}}. \end{array}$$

The six linear independent equations in the simultaneous Equation (23) are solved and substituted into the quadratic polynomial. The axial deformation function of the half-model of the rectangular section:

(56) $$\begin{array}{c} \psi_{\varphi 10}^{\chi_{b}}\left(s\right)=\left\{\begin{array}{cc} -\frac{2}{h}\left(s-s_{AD}\right)+1, & s\in \left[s_{AD},s_{AB}\right),\\ \frac{2}{b}\left(s-s_{AB}\right)-1, & s\in \left[s_{AB},s_{BC}\right),\\ -\frac{2}{h}\left(s-s_{BC}\right)+1, & s\in \left[s_{BC},s_{CD}\right),\\ \frac{2}{b}\left(s-s_{AD}\right)-1, & s\in \left[s_{CD},s_{DA}\right). \end{array}\right. \end{array}$$

**Figure 19:**
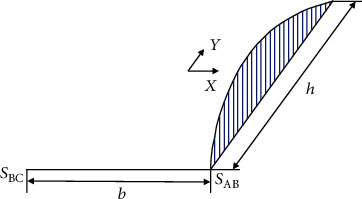
Bending warping section deformation.

According to the warping deformation characteristics and geometric symmetry characteristics of the warping deformation, the bending warping model of the section is simplified to be solved by the half-model as shown in Figure19.

**Figure 20:**
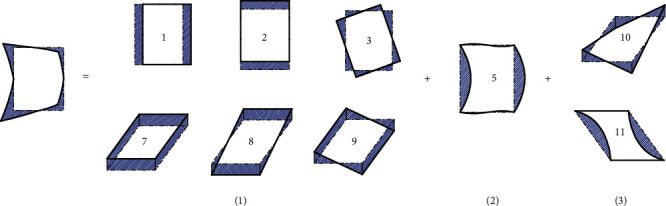
Deformation mode and its composition.

According to the half-model shown in Figure20, the following six conditions are used as boundary conditions for determining the plane profile function of the two side wall plates of the corner node:

(1) The deformation on wall *A* is symmetrical about the center point of wall *A*, and the slope of the axial deformation function at the symmetry point is 0:
  (57) $$\begin{array}{c} {\left.\frac{d\psi_{\varphi 11}^{\chi_{b}}\left(s\right)}{ds}\right|}_{s_{AD}+h/2}=0. \end{array}$$
(2) The axial deformation of wall *B* is 0:
  (58) $$\begin{array}{c} {\left.\psi_{\varphi 11}^{\chi_{b}}\left(s\right)\right|}_{s_{AB}}=0. \end{array}$$
(3) The amplitude of the center point of wall *A* shown in Figure 12 is 1:
  (59) $$\begin{array}{c} {\left.\psi_{\varphi 11}^{\chi_{b}}\left(s\right)\right|}_{s_{\text{AD}}+h/2}=1. \end{array}$$

The six linear independent equations are combined in the joint solution, solved, and substituted in the quadratic polynomial. According to the symmetry characteristics, the axial deformation function of the model is obtained:

(60) $$\begin{array}{c} \psi_{11}^{\chi_{b}}\left(s\right)=\psi_{\varphi 11}^{\chi_{b}}\left(s\right)=\left\{\begin{array}{cc} -\frac{4}{h^{2}}{\left(s-s_{AD}\right)}^{2}+\frac{4}{h}\left(s-s_{AD}\right), & s\in \left[s_{AD},s_{AB}\right),\\ 0, & s\in \left[s_{AB},s_{BC}\right),\\ \frac{4}{h^{2}}{\left(s-s_{BC}\right)}^{2}-\frac{4}{h}\left(s-s_{BC}\right), & s\in \left[s_{BC},s_{CD}\right),\\ 0, & s\in \left[s_{CD},s_{DA}\right). \end{array}\right. \end{array}$$

The above is the expression of the shape function of each feature deformation mode. After substituting the above equation into the experimental structure, each coefficient *χ*_*b*_^*L*^, *χ*_*b*_^*D*^, and *χ*_*b*_^*W*^ for each section is solved and returned to the original equation to obtain the values. The effects of distortion and warping in the low- and high-order modes under the action of the thin-walled curved beam under the in-plane load are clearly shown in Figure20.

## 6. Results

As the experimental results show, the values of 60 positions in the walls are as shown in Table 3. The strain at the test position of wall *A* is negative, that is, a compression value, while those at the locations of wall *C* are positive, or tensile, the value of 2 position approaches 0. The value of *B* and *D* walls near wall *A*, that is, the 3 locations in wall *B* and 1 position in wall *D* are positive values, that is, tensile, while the far wall *A* is negative, and the value of the 2 position point is the middle value of the 1 position point and the 3-position point value.

**Figure 21:**
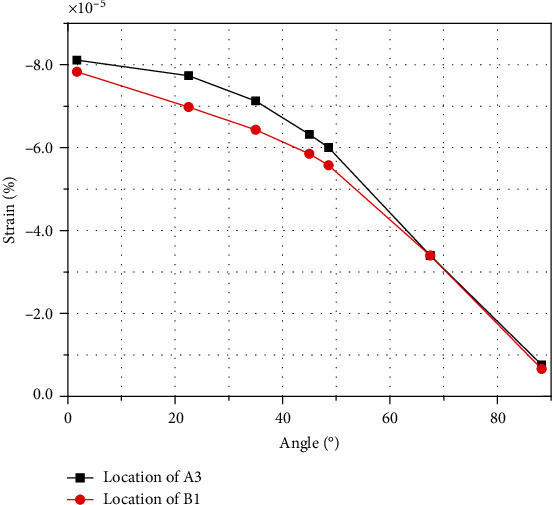
Test deformation varies of location

Taking a typical 3-position point in wall A and 1-position point in wall B as examples, the strain change of the entire curved beam is represented. The value is as shown in Figure21. It also can be seen from the figure that the strain value of locations in compression and tensile of the curved beam are basically the same; the values decrease from the constrained end to the free end, which conforms to the trend of force deformation.

**Figure 22:**
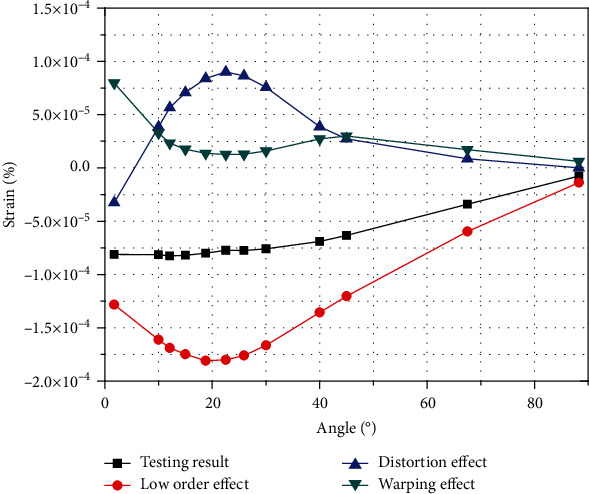
Low-order, distortional, and warping effects.

Figure22 shows the results of the experimental test A3 position and the results of the one-dimensional experimental test theory. The graph compares the actual deformation with the low-order, distortion, and the warping effects.

The low-order effect is the main influencing factor of the deformation which is marked by red line, and the influence value is larger than those for distortion and warping. The low-order effect value becomes larger and then smaller, and the maximum value occurs at the second test point of 22.5° which finally approach 0.

The distortion effect is mostly compression amount which is marked by blue line, for tensile effect; the value changes from large to small and becomes larger after approaching 0. The maximum value also occurs at the 22.5° test position, before decreasing again and approaching 0. In warping marked by green line, the influence has a positive value, that is, the tensile effect. The influence value first changes from large to small. The minimum extreme point also occurs near the 22.5° position; the value then increases to the maximum extreme point at 45° test position and then decreases until it approaches 0.

**Figure 23:**
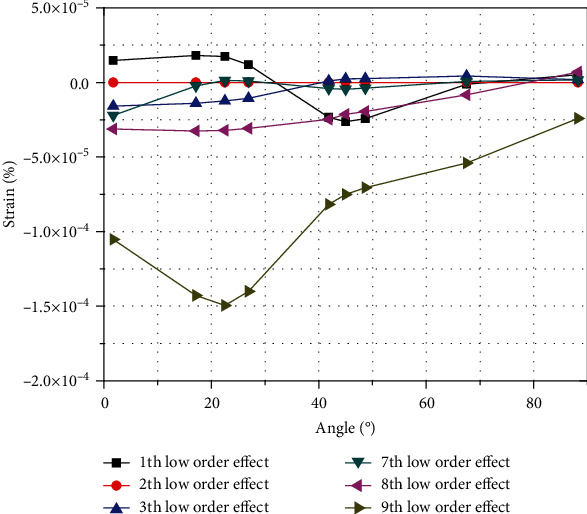
Six low-order effects.

For low-order influence, the above actual deformation value derivation and one-dimensional test theory can obtain the deformation form and its composition. The respective influences of various low-order characteristic deformations under the action of in-plane loading are illustrated in Figure23, with the low-order effects obtained after calculation *u*_*L*1_(*s*_*j*_, *ϕ*),  *u*_*L*2_(*s*_*j*_, *ϕ*), *u*_*L*3_(*s*_*j*_, *ϕ*), *u*_*L*7_(*s*_*j*_, *ϕ*), *u*_*L*8_(*s*_*j*_, *ϕ*), and *u*_*L*9_(*s*_*j*_, *ϕ*). The influences of the six low-order strains show that the low-order deformation, except the ninth characteristic deformation mode, is near 0, and the influences are smaller than that of the ninth characteristic deformation mode. In the low-order deformation effect, the ninth characteristic deformation mode has the greatest influence, which means that the displacement clockwise along the *y*-axis is the largest. This direction is consistent with the direction of load application, which determines the low-order influence throughout the deformation, with a trend matching the overall low-order effect.

The first deformation mode is translation along the *x*-axis, and the deformation from the constrained position to 32° along the curved beam is in the direction of the outer diameter along the *x*-axis. From this area to the load application position, the negative direction along the *x*-axis is the reverse translation of the inner diameter, and the influence value changes from small to large. The maximum extreme point occurs at 45° and then becomes smaller until it approaches zero. The second characteristic deformation mode of low-order deformation has no effect, that is, the curved beam under the in-plane load has no translation along the *y*-direction. The third and seventh deformation modes have little influence, that is, rigid rotation along the *z*-axis and the axial expansion are both negative and gradually approaching zero. Rigid rotation around the *x*-axis (the eighth deformation mode) is clockwise, and the influence value changes from large to small and finally approaches zero.

**Figure 24:**
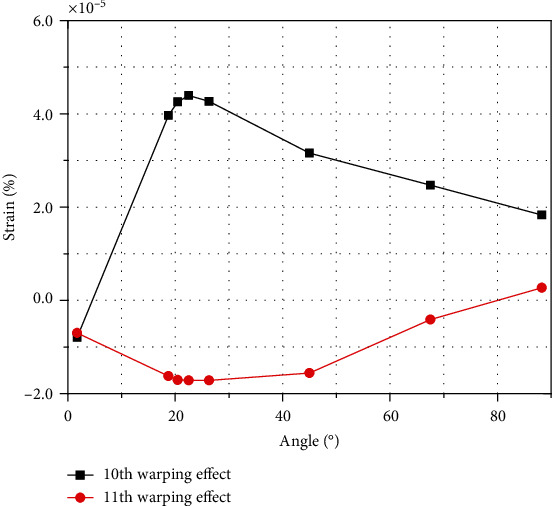
Bending torsional warping effects.

The 10th deformation mode in the warping effect is the torsional warping effect, and the 11th mode is the bending warping effect.  Figure24 shows that the two warping forms affect the curved beam under in-plane loading, the torsional effect is greater than the bending effect, and both vary from large to small, with the maximum extreme points occurring at the 22.5° test point.

## 7. Conclusion

A new method of one-dimensional deformation experimental test theory has been proposed for calculating and judging the contributions of low-order deformation effect and the high-order distortion and warping effects to the actual deformation of a thin-walled curved rectangular box beam under in-plane bending loads. Because the vector of deformation cannot be directly measured, we converted vectors in three directions containing axial, tangential, and normal deformations into superimposable tensile and compressive amounts, which are scalars that can be superimposed and matched with experimental data. As to the finite element analysis of plane problem on curved beam, four nodes triangular element performs better property on higher accuracy and faster convergence, then the deep collocation method element; more neurons and hidden layers could obtain better results, while the inverse finite element method experimentally measured strains are used as input data, in which more strain gauges should be used. This theory can be implemented in elastomeric materials and structures to measure the deformation distribution of various elastomer materials and structures and other mechanical properties of the structure. An experimental test system was established to obtain the deformation distribution of the entire curved beam structure at any position. The deformation value of the entire curved beam structure under the bending load was determined by marking 60 test locations evenly distributed on the four walls at five cross-sectional locations on the curved beam structure. The significance of the distortion and warping components in the actual deformation and the main role of low-order deformations are verified. The magnitude and variation trends of the eleven deformation modes are obtained; no radial translation of the curved beam structure occurs under in-plane bending.

## Figures and Tables

**Table 1:** Normalized tip deflection of the thin curved beam.

Mesh density			1 × 6	2 × 12	4 × 24
Element type	Node	Degree of freedom	Normalized deflection		
Quadrilateral elements					
RY-Q10	5	10	-0.08901	-0.08844	-0.08846
RY-Q14D	5	14	-0.08748	-0.08898	-0.08925
RY-Q18	9	18	-0.08745	-0.08840	-0.08850
Triangular elements					
RGR-T10	5	10	-0.06305	-0.08493	-0.08609
RGR-T11D	7	11	-0.08291	-0.08434	-0.08691
RGR-T10D	4	10	-0.06486	-0.08501	-0.08650
Standard reference value is 0.08734^∗^					

**Table 2:** Maximum deflection predicted by deep collocation method.

Clamped square plate	Predicted maximum deflection			
Number of hidden layers	20 neurons	30 neurons	40 neurons	50 neurons
1	0.860568	1.152175	1.195843	1.24987
2	1.257226	1.257759	1.265145	1.261974
3	1.26178	1.260374	1.261743	1.258893
Exact solution is 1.260000^∗^				

**Table 3:** Variation in test deformation in the entire curved beam.

Wall	Section	1			2			3			4			5		
	Position	1	2	3	1	2	3	1	2	3	1	2	3	1	2	3
A	Sign	—	—	—	—	0	—	—	0	—	—	0	—	—	0	—
B		—	+	+	—	+	+	—	+	+	—	+	+	—	0	+
C		+	+	+	+	0	+	+	0	+	+	0	+	+	0	+
D		+	—	—	+	—	—	+	—	—	+	—	—	+	—	—

## Data Availability

The data used to support the findings of this study are available from the corresponding author upon request.
